# Factors influencing colorectal cancer screening decisions among Saudi women: A qualitative study

**DOI:** 10.1371/journal.pone.0321086

**Published:** 2025-04-07

**Authors:** Norah Alsadhan, Cathy Brennan, Sultana A. Alhurishi, Farag Shuweihdi, Robert M. West

**Affiliations:** 1 Leeds Institute of Health Sciences, School of Medicine, University of Leeds, Leeds, United Kingdom; 2 Psychological & Social Medicine, School of Medicine, University of Leeds, Leeds, United Kingdom; 3 Department of Community Health Sciences, College of Applied Medical Sciences, King Saud University, Riyadh, Kingdom of Saudi Arabia; 4 Dental Translational & Clinical Research Unit, School of Dentistry, University of Leeds, Leeds, United Kingdom; Virginia Mason Franciscan Health, UNITED STATES OF AMERICA

## Abstract

**Background:**

Colorectal cancer (CRC) is a major global health challenge and one of the most prevalent cancers in Saudi Arabia. Studies show that young Saudi women are often diagnosed with CRC at more advanced stages, leading to poorer prognoses. Despite the recent launch of the first Saudi national CRC screening program, public awareness and acceptance of CRC screening (CRCS) remain limited.

**Methods:**

We conducted semi-structured interviews with 17 women aged 40 or older to explore their awareness, views, and attitudes toward CRC and CRCS. Data were analyzed using reflexive thematic analysis, and the Social Ecological Model was applied to guide the structuring and organization of the developed themes.

**Results:**

We identified a multifaceted interplay of knowledge, beliefs, and social-cultural factors influencing CRCS decisions among Saudi women. Although there was a general awareness of CRC, understanding of risk factors, signs, and symptoms was limited. Many participants adopted a reactive approach to screening, prompted by symptom manifestation or family history, rather than preventive health measures. Concerns such as fear of a cancer diagnosis and discomfort with the stool sample collection process hindered screening participation. Social support from family and community, and physician recommendations were crucial in encouraging screening uptake. Logistic and digital literacy challenges in accessing health services were noted for older adults. Participants stressed the need for increased CRC awareness, equitable access to screening services, and reminders to improve CRCS participation.

**Conclusion:**

Factors influencing CRCS uptake among Saudi women are complex and multifaceted. Comprehensive and tailored health promotion interventions that meet community needs are essential. Further research is needed to develop and evaluate the effectiveness of these interventions in increasing screening uptake.

## Introduction

Colorectal cancer (CRC) is a major global health challenge and one of the leading causes of cancer-related deaths worldwide [1]. In high-income countries, CRC rates among individuals over 50 years have stabilized, mainly due to effective screening programs [2,3]. However, there is a concerning rise in early-onset CRC (EO-CRC), affecting individuals < 50 years [4]. In 2020, CRC was the most commonly diagnosed cancer among males and the third among females in Saudi Arabia, with 1,729 new cases representing 12.3% of all new cancer diagnoses [5]. Over the past three decades, the incidence of CRC in Saudi Arabia has steadily increased across all age groups [6,7]. According to the 2019 Global Burden of Disease (GBD) report, Saudi Arabia recorded the highest annual percentage change in EO-CRC incidence rates among all included countries [8]. EO-CRC tends to be more aggressive, thus presenting a considerable health burden for young adults [9].

The prognosis of CRC varies according to the disease stage at diagnosis [10]. Early diagnosis of CRC leads to better treatment outcomes, enhanced survivorship, reduced healthcare costs, and improved patient quality of life [11,12]. In 2020, approximately one-third of Saudi CRC cases were diagnosed late with distant metastasis [5]. Sex disparities in the CRC stage at diagnosis have been reported in the Saudi literature. A 2015 cohort study of 1016 Saudi CRC patients found that women were 20% more likely than men to present with a metastatic tumor [13]. A recent retrospective cohort study of 17,541 CRC patients from the Saudi Cancer Registry indicated that young ( < 50 years) women had an increased risk of late-stage CRC [14]. Additionally, Zacharakis et al. noted a female predominance in EO-CRC among participants aged 45–50 in the Al-Kharj pilot screening program conducted from 2017 to 2022 [15]. These findings underscore the need for further research to elucidate the underlying causes contributing to late-stage diagnoses in females. Additionally, tailored interventions to enhance early detection rates among this population are warranted.

CRC screening (CRCS) has been shown to decrease the incidence and mortality rates associated with CRC in the general population [16]. In line with the Saudi 2030 vision, the Ministry of Health (MOH) launched the first Saudi National CRC screening program in 2017. Based on the individual’s risk, this program provides screening services such as fecal occult blood tests and colonoscopies. The program operates across the kingdom’s 13 administrative regions through participating primary healthcare centers [17]. Average-risk individuals- those who are asymptomatic and aged between 45 and 75- are advised to undergo annual Fecal Immunochemical Test (FIT) screening. Participants are instructed to collect a stool sample using a provided container and return it to the healthcare center for analysis. If the FIT results are positive, a follow-up diagnostic colonoscopy is recommended.

The MOH’s screening program is currently the only initiative targeting the general Saudi public. To our knowledge, there is limited data on public awareness, participation, and attitudes towards this program. Additionally, publicly available reports on program metrics such as attendance rates, adherence, and patient outcomes are lacking.

A recent report on the Al-Kharj pilot CRC screening program, conducted exclusively in the Al-Kharj city, showed a high participation rate of 73% over five years. Data on participation rate by sex are not available [15]. In 2023, Almadi and Basu identified various operational challenges in CRCS implementation in Saudi Arabia that warrant attention. They highlighted the need to address issues such as workforce shortages, limited coordination between healthcare facilities, the absence of a quality assurance system to monitor the program, and inefficiencies in system design that negatively impact the patient’s journey [18]. Additionally, a recent review of CRCS challenges in Saudi Arabia indicated two major concerns relating to low physician recommendation rates and limited participation among females [19].

Knowledge and perceptions about cancer and screening practices can influence the decision to participate in CRCS [20,21]. Although several studies have examined the knowledge and acceptance of CRC among the Saudi population, findings consistently show low CRC awareness and screening uptake [22,23]. A nationwide survey in 2019 revealed that males were more likely to accept CRCS than females [24]. However, an in-depth exploration of Saudi women’s views on CRCS is lacking. The existing literature on CRCS awareness in Saudi Arabia predominantly comprises survey-based studies. These studies, while informative, do not explore the deeper perceptions and attitudes that influence CRCS uptake. Thus, qualitative research is needed to capture the complexities of personal and cultural beliefs that structured surveys might not fully address [25].

We aim to explore Saudi women’s awareness of CRC and their perceptions and attitudes toward CRCS. These insights are crucial for enhancing national screening efforts, tailoring public health promotion strategies, and improving CRC outcomes among women in Saudi Arabia.

## Methods

### Design

We conducted semi-structured one-to-one interviews with participating women. The Consolidated Criteria for Reporting Qualitative Studies (COREQ) guided the conduct of the study and the reporting of findings [26]. This study was approved by the Research Ethics Committee of the School of Medicine at the University of Leeds (MREC 22–101) and by the Ethics Committee of King Saud University (KSU-HE-23–680).

### Study participants

We recruited female employees aged 40 or older from King Saud University, Saudi Arabia. To capture diverse perspectives and experiences, we used purposive sampling to recruit individuals with varying ages and educational backgrounds. To further enhance diversity, snowball sampling was used, where initial participants were encouraged to refer individuals of different ages and education levels. Women who were currently under investigation or diagnosed with any cancer were excluded. The recruitment process, coordinated by (NA), began with a study advertisement emailed to all potential participants. Interested participants were notified via follow-up emails that the invitation process might be delayed to prioritize diversity and that not all who expressed interest would be selected. Interviews were scheduled at the convenience of the participants.

Based on the objectives of this study, we initially aimed to recruit a minimum of 10 participants, focusing on a diverse sample of age and educational levels. The decision regarding sample size was not driven by the traditional concept of data saturation; instead, it was based on pragmatic considerations such as time constraints and the principle of information power [27]. This principle underscores the importance of iterative evaluation during data collection, ensuring that the depth and quality of data obtained from interviews were sufficient to address the research questions meaningfully [28]. Given our use of reflexive thematic analysis in this study, these considerations proved more appropriate for guiding our approach to sampling [27].

### Data collection

(NA) and (CB) developed a semi-structured interview guide after a thorough review of the literature [29–32]. The guide included open-ended questions that explored women’s health-seeking behavior, awareness of CRC and CRCS, and perceptions about the benefits, barriers, and facilitators of CRCS. The guide was piloted with two women and amended according to their feedback.

Participants received an information sheet and completed a written consent form before the interview. They all chose to be interviewed via Microsoft Teams with the video feature turned off. The interviews were conducted from October 17, 2023 to November 26, 2023 by (NA), a researcher with training and expertise in qualitative research methods. Participants were asked about their general knowledge of CRC and CRCS at the beginning of the interview. The CRC National Screening Program was then explained using infographics published by the MOH [17]. Participants then shared their perceptions of the benefits, barriers, and facilitators influencing their decision to undergo screening. All interviews were conducted in Arabic, audio-recorded, and transcribed verbatim. Once transcription was completed and verified, all audio recordings were deleted. To ensure confidentiality, all transcripts were anonymized and pseudonymized, with participants identified solely by assigned numbers and initials. Any identifiable information, such as names of individuals or institutions, was also pseudonymized. Transcripts were labeled using each participant’s assigned pseudonym initials and the interview date. All research data, including transcripts, audio recordings, and consent forms, were securely stored in a password-protected folder on the lead researcher’s university-provided OneDrive account, with access restricted to the research team.

### Data analysis

Our analysis was conducted in two stages. First, we used Reflexive Thematic Analysis (RTA), a theoretically flexible method for analyzing qualitative data to explore participants’ experiences and perceptions. Braun and Clarke [33] outlined six main phases for RTA: familiarization with the data, generation of initial codes, searching for themes, reviewing themes, defining and naming themes, and producing the final report. A core principle of RTA is its acknowledgment of the researcher’s subjectivity and assumptions as valuable resources for generating knowledge. Unlike other thematic analysis approaches, RTA discourages using structured codebooks or reliability measures. Instead, it adopts an iterative and interpretative reflexive approach to analysis, where coding evolves organically through the researcher’s active engagement with the data [34].

In the first stage, the lead author (NA) became familiar with the data by repeatedly listening to the audio files and reading the interview transcripts. Following familiarization, NA began coding all transcripts using MAXQDA software (Version 24) [35]. An inductive approach to coding was adopted, allowing the development of codes and themes directly from data without the constraints of predefined or existing theoretical frameworks [34]. Both semantic coding, which focuses on the explicit meaning of data in relation to the research questions, and latent coding, which identifies underlying assumptions and implicit meanings, were employed [34].

To enhance the depth and richness of the analysis, another author (SA) contributed to the coding process by double-coding a sample of transcripts. This collaboration aimed to discuss and refine initial thoughts about the data, not to ensure accuracy or reliability, as such practices are not aligned with RTA principles [36]. Coding was iterative, with regular team meetings (NA, SA, and CB) to review, refine, and collate codes. Codes with similar meanings were grouped together to develop initial themes, which were iteratively reviewed and refined by the research team. NA and SA iteratively reviewed the transcripts to ensure that the thematic structure accurately represented coherent patterns of meanings within the data. All authors jointly reviewed and discussed the findings and agreed on the final themes reported in the study.

Upon reviewing these themes in the second stage, we determined that the Social Ecological Model (SEM) provided a useful framework to further structure our analysis [37]. According to the SEM, health decisions like participating in CRCS are influenced not only by personal factors but also by various determinants at multiple levels—individual, interpersonal, organizational, community, and policy [38]. We aligned our identified themes with these SEM levels to refine our interpretation of the findings. This alignment is crucial for developing targeted interventions that address multiple factors, potentially increasing CRCS uptake among women. The lead author (NA) mapped these themes to the SEM levels, which all authors reviewed. The quotes presented in the findings were translated from Arabic to English by (NA) and verified by (SA).

## Results

### Participant characteristics

**Fig 1:**
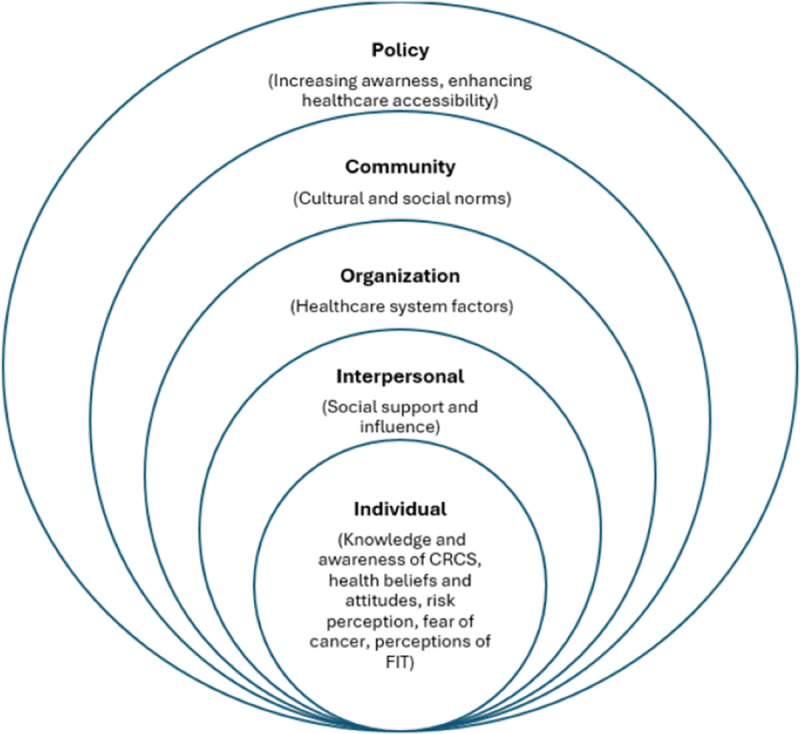
A Social Ecological Model illustrating levels of influence and factors affecting CRCS decisions.

We interviewed 17 women to explore their views on CRC screening (Table 1). The participants represented diverse age groups and educational backgrounds. While none of the women reported having a personal history of cancer, eleven (65%) disclosed a family history of the disease, with four mentioning a history of CRC in their families. Interview duration ranged from 16 to 42 minutes. Findings were organized into ten themes mapped onto the five levels of the SEM: individual, interpersonal, organizational, community, and policy (Table 2 and Fig 1).

**Table 1:** Participant’s characteristics.

Participant	Age	Educational level	Family history of cancer	Family history of CRC
1	40	PhD	No	No
2	41	Master	Yes	Yes
3	42	PhD	Yes	No
4	43	Bachelor	Yes	No
5	67	Bachelor	Yes	No
6	50	PhD	No	No
7	55	High school	Yes	No
8	63	High school	No	No
9	47	Bachelor	Yes	No
10	45	Bachelor	Yes	Yes
11	62	PhD	No	No
12	46	Bachelor	No	No
13	53	Master	Yes	No
14	57	PhD	Yes	Yes
15	48	Bachelor	No	No
16	53	Master	Yes	Yes
17	45	PhD	Yes	No

Note: CRC: colorectal cancer.

**Table 2:** Factors influencing CRC screening uptake among Saudi women.

1. Individual-level factors	2. Interpersonal-level factors	3. Organizational-level factors	4. Community-Level Factors	5. Policy-Level Factors
1.1 Knowledge and awareness of CRCS*:	2.1. Social support and influence*:	3.1. Healthcare system factors*:	4.1. Cultural and social norms*:	5.1. Increasing awareness*:
Awareness gaps	Influence of family and friends on motivation/ decision	Influence of physicians	Cultural attitudes towards screening	Comparison with other successful health promotion campaigns
Misunderstandings	The dual impact of family influence	Holistic and proactive healthcare approach	Word-of-mouth influence	Health education topics
Influence of personal/family experience		Satisfaction with the primary healthcare services	Role of social influencers and community leaders in promoting health	Use of multiple media channels
1.2. Health beliefs and attitudes*:		Logistical and practical challenges to seeking care	Religious beliefs	Use of official information channels
Importance of early detection		Sterilization and hygiene concerns		Effective and tailored education
Priority of health		Special consideration for older adults		Influence of storytelling
Self-efficacy				5.2. Enhancing healthcare accessibility*:
1.3. Risk perception*:				Uniform availability of screening
Symptom-driven testing				Access in remote and rural areas
Misconceptions				Automated reminders and government platforms
Influence of family history				
1.4. Fear of cancer* (fear as a barrier/motivator)				
1.5. Perceptions of FIT*:				
Aversion to sample collection (embarrassment, disgust)				
Test administration				
Accuracy concerns				
Acceptance				
Physical discomfort				

Note: CRC, colorectal cancer; CRCS, colorectal cancer screening; FIT, fecal immunochemical test.

*Indicates key themes within each SEM level, with bullet points summarizing the main findings under each theme.

## Factors influencing CRCS decisions

### 1. Individual level factors

#### 1.1. Knowledge and awareness of CRCS.

When asked about preventive health practices, most women expressed general awareness of cancer screening. However, they were unfamiliar with the screening tests for CRC or the targeted age group. Many women noted that despite their general awareness of CRC, they were uncertain about its risk factors, signs, and symptoms. Some women attributed their knowledge deficit to the perceived low prevalence of CRC in the Saudi community.

*“We hear about it, but I don’t know exactly what symptoms or pains the patient might feel. I hear a lot about it, but I don’t know what specific tests are involved.”* P12

*“We lack awareness about these issues because, fortunately, they are not prevalent in our community.”* P10

Several participants seemed confused by medical terminology and concepts, such as the meaning of “family history,” and demonstrated a lack of understanding regarding basic human anatomy.

*“We hear about colon disorders and irritable bowel syndrome, but colon cancer doesn’t come to mind. I mean colon cancer, I don’t know, it doesn’t come to mind because they always say the colon isn’t an organ, so it’s surprising when they mention colon cancer.”* P12

Personal and community experiences seemed to influence an individual’s understanding of CRC. For example, women who had undergone CRCS, had a family history of CRC, or knew others in the community affected by the disease seemed to have a better understanding and provided details about treatment. However, some still reported a lack of knowledge.

*“Honestly, I don’t know much about it, but because we [the family] had a case of death from it, I have heard about it. However, I have no idea about its symptoms or anything like that.”* P10

#### 1.2. Health beliefs and attitudes.

Most participants recognized the importance of early CRC detection for cancer prevention, better treatment outcomes, and higher survival rates. Women also underscored the psychological benefits of screening, emphasizing the mental relief it provides.

*“Certainly, if the doctor tells me to get tested, God willing, I will do it. First and foremost, it’s to reassure myself about my health and to understand my medical condition. Secondly, it helps me feel mentally at ease. Getting the tests done means that if anything is detected early, I expect the treatment will be easier and better than if it were discovered in later stages.”* P12

Several women highlighted the connection between personal health and family well-being. They discussed the necessity of prioritizing health due to familial responsibilities and expressed concern about the emotional and psychological impact their potential illness could have on their loved ones.

*“You are helping yourself and those around you from potentially losing you. They might not experience physical pain like you, but they will endure psychological pain and the fear of loss.”* P14

Despite recognizing the benefits of health screenings, several participants expressed that balancing these practices with other life demands can be challenging. This reflects how health is often prioritized and valued relative to other responsibilities. However, one woman with high self-efficacy expressed strong determination and a proactive approach to overcome these barriers.

*“People are preoccupied with their families, homes, and daily lives, so they don’t remember.”* P5

*“I’ll do anything to find the right time to do the test. What occupies me the most is my job and work hours, but I’ll do anything to make the time to go and do it.”* P13

#### 1.3. Risk perception.

Participants reacted differently to information about the CRC screening program. While some showed interest in participation, many demonstrated a conditional approach to preventive health care, often weighing the perceived necessity of screenings against their current health status. This ambivalence reflects a broader reactive trend, where the absence of immediate symptoms diminishes the perceived urgency for regular checkups and tests.

*“Sometimes I think I want to go and get checkups and tests done, but other times I think, as long as I’m thankfully healthy and well, there’s no need.”* P12

*“If I were sick and feeling bad, I’d do everything necessary, but as a preventive measure, I’m not sure.”* P17

Misconceptions about the risk of CRC also influenced decisions to undergo screening. Several participants believed CRC was uncommon in their community or predominantly affected men. These perceptions foster a false sense of security and diminish the perceived need for proactive health measures.

“*This is quite common among men, actually. I know of two men who have died from this issue. Thankfully, there haven’t been any cases among the women.”* P10

Family history seemed to impact participants’ perceptions of personal risk and their motivation to engage in screening. Women with a family history of CRC or other cancers perceived an increased risk of CRC and recognized the importance of preventive actions. Conversely, participants without a CRC family history felt less urgency about screening; however, some acknowledged that if a hereditary risk were present, they would be more encouraged to undergo screening.

*“In our family, colon cancer is present among the men, so as a preventive measure, I wanted to check myself. I didn’t have any symptoms or anything; I just wanted to be proactive.”* P2

*“The first thing that encourages me to get checked is having a CRC family history, which means there’s a hereditary factor. This means I could potentially be affected, especially if it’s among first-degree relatives.”* P4

#### 1.4. Fear of cancer.

Fear of cancer seemed to influence the decision-making process regarding CRC screening, affecting individuals regardless of their family history of cancer. Yet, fear appeared to operate differently across individuals, affecting their screening behaviors in diverse ways. For some, fear acts as a barrier; the dread of uncovering an illness can provoke anxiety, leading to avoidance behaviors aimed at escaping potential bad news. Conversely, others view fear as a motivator, compelling them to engage in proactive health behaviors. A woman with a family history of breast cancer normalized the fear associated with screening and viewed it as an impetus for action.

*“Everyone is naturally afraid of disease, but I acknowledge my fear of disease and want to detect it early. I will go and get tested so that, God forbid, I can protect myself, and if, God forbid, there is cancer, I can take control of the situation early.”* P13

#### 1.5. Perceptions of FIT.

Most women expressed embarrassment and disgust regarding the collection and storage of stool samples, viewing this process unfavorably compared to other types of medical specimen collection. Furthermore, some women expressed concerns about the FIT administration, particularly regarding correctly collecting the stool sample. These concerns were especially pronounced when considering older adults.

*“If I were to give the test instruments to my father, I’d be doubtful he knows how to do it. My father has a tremor, and he’s old. I feel there’s no way he would know how to make the correct scratch. So, I won’t trust the results when they come out.”* P1

Several participants expressed reservations about the reliability of the FIT, citing concerns about the potential for false negative or positive results. This led some to state a preference for professional testing in a clinical setting, believing it would yield more accurate and dependable outcomes. Others felt that a colonoscopy, despite its invasive nature, would provide more comprehensive and precise results than FIT.

*“I feel more comfortable going to the hospital to do it. I feel it will be more accurate. At least if I do it at the hospital, I’m sure the result will be 100% accurate.”* P4

*“If someone is afraid they might have colon cancer, performing a colonoscopy is more accurate and comprehensive, whether there is blood or not.”* P1

Despite these concerns, many participants felt the test was convenient and straightforward. The non-invasive nature of FIT was particularly appreciated, as it avoids the physical discomfort, fear, and embarrassment often associated with other screening methods. The ability to perform the test in the privacy of one’s home was also highlighted as an advantage. However, one woman expressed a general worry about the physical discomfort associated with screening procedures, which deterred her from participating even before learning about the testing process. These worries were often based on anecdotal experiences shared by others.

*“Sometimes, fears can prevent you from getting screened. You know, you hear from people that the breast cancer screening is painful and hurts, so now I’ve just decided to cancel the idea of early screening.”* P15

### 2. Interpersonal level factors

#### 2.1. Social support and influence.

The experiences and behaviors of individuals within one’s social network can potentially shape health behavior decisions. Several women expressed that observing collective participation in CRC screening acts as positive reinforcement, reducing anxiety around the procedure, normalizing the behavior, and ensuring its effectiveness. Others emphasized that personal connections and emotionally impactful experiences motivate individuals to prioritize and participate in health screenings. Without such connections, screening can seem irrelevant.

*“If you see many people getting tested, you feel encouraged and reassured that the screening is beneficial.”*P7

*“I don’t know anyone personally affected by it, and sometimes there are experiences that touch you deeply. This might be one of the reasons why you don’t feel the need to get it done.”* P6

Additionally, having concerns about family members’ health seemed to motivate individuals to seek information and consider preventive screenings. This motivation is often driven by a deep care for loved ones and a strong desire to manage potential health risks within the family.

*“My husband has Crohn’s disease, which affects the intestines. Given that he is among those at increased risk, could it be possible, God forbid, that he might also need to be screened for colon cancer?”* P12

Family context appeared to play a crucial role in shaping personal health decisions, particularly concerning sensitive topics like the stool collection process for CRCS. The degree of openness within the family may influence the support participants receive. For example, one woman expressed her discomfort in discussing such matters with her spouse:

*“We are now in a formal meeting, but if it’s my husband, I would feel shy to say to him let’s go to deliver the sample, really I would feel shy.”* P14

Open discussions about health concerns within the family seemed to have a dual impact on an individual’s healthcare actions. While some participants felt that family involvement often acts as a barrier—discouraging medical visits or downplaying symptoms— others reported that robust family support encouraged them to take proactive health measures.

*“Convincing my family that I am going to get this screening is a hurdle. Sometimes they say, ‘No, what for? You don’t need it, you don’t have symptoms, you are fine.’ Yet, I cannot go without informing them.”* P16

*“I consult with my husband, and then, yes, I go. He is usually firm and tells me it’s best to see a doctor. I tend to be more relaxed about it.”* P5

### 3. Organizational level factors

#### 3.1. Healthcare system factors.

Most women acknowledged the critical role of physician recommendations in guiding their health decisions, including their participation in CRCS. They expressed trust in their doctors, especially when there was an established relationship, believing that medical advice was given in their best interests. This trust often extended to familiar healthcare settings, which seemed to enhance adherence to medical advice.

*“I want to continue with my doctor and hospital. I don’t want to go to a place where it feels like starting all over again.”* P14

However, this trust did not universally translate into confidence in the broader healthcare system. Some participants voiced concerns about the system’s reactive nature and questioned whether healthcare providers were adequately informed about preventive measures. These concerns led to calls for systemic changes towards a more holistic and proactive model of care, including integrating CRCS into routine checkups across various specialties.

*“Doctors, such as family physicians, general practitioners, or doctors in general, should encourage their patients by saying, “It’s best for you to begin colorectal cancer screening now.” Our issue is that we lack a holistic approach.”* P17

Most women expressed satisfaction with the primary healthcare services, highlighting that they are free and praising the widespread availability and accessibility of health centers. They believed these qualities positively influence women’s decisions to undergo screening.

*“It’s convenient that the health center can receive the samples. It’s a suitable solution because it’s close to home, and one can stop by on their way to or from work.”* P16*.*

However, not all participants viewed the process of seeking healthcare positively. Some women perceived seeking care as burdensome due to transportation challenges, such as long distances and crowded streets. These difficulties potentially discourage regular visits and necessary checkups. Several women also stated frustration with operational inefficiencies at health centers, particularly noting the crowding and long waiting times. Many women suggested establishing mobile clinics in high-traffic areas such as universities, shopping centers, or workplaces to encourage participation without needing scheduled appointments.

*“The ease of access was key; I didn’t need to go to the hospital or make an appointment. I was heading to the mall and could do it easily when I found it [screening clinic] right before me.”* P5

Some women highlighted their mistrust of the protocols and practices that ensure the cleanliness and safety of medical equipment. Concerns about potential contamination from inadequately sterilized tools deterred their acceptance and participation in CRCS.

*“People are afraid that, God forbid, the tools might not be sterilized, for example, or they fear that the virus could be transmitted to them or something like that.”* P7

Many women emphasized the importance of respecting societal values of cleanliness, suggesting the need to create suitable conditions for storing the sample. Providing specialized containers for sample storage and enabling direct delivery to clinics could address hygiene concerns and make the screening process more acceptable.

*“We are a very clean society; you must create suitable conditions for storing the sample by any means necessary. If there are no alternatives, people could bring it directly to the clinic’s location instead of storing it in a home fridge.”* P6

Several women highlighted the challenges older adults face in navigating healthcare, including transportation issues and the lack of appropriate equipment for those with physical limitations. They emphasized the need for more flexible healthcare service hours, such as afternoon or evening clinics, to accommodate older adults’ varying schedules. Additionally, reliance on digital platforms for scheduling appointments presented another obstacle due to digital literacy barriers. To enhance access for the elderly, several women recommended expanding home-based health services to include CRCS.

*“Just as you [health institutions] provide services where they [healthcare personnel] go and take the tests at home, take them [CRC test kits] and go to their [the elderly] homes to do it for them.”* P10

### 4. Community level factors

#### 4.1. Cultural and social norms.

Women discussed a pervasive mindset within the community that prioritizes reactive health care measures over proactive ones, leading to general neglect of preventive health practices regardless of an individual’s educational background or personal knowledge.

*“We’re talking about a community; I’m speaking about the world around me, whether educated or not. We tend to exhibit laziness, neglect, and indifference. Take my husband as an example—he’s a well-educated, cultured man who studied abroad. However, when it comes to his health or visiting hospitals, he won’t go unless forced. He views regular health checks as an obsession or madness.”* P13

Participants emphasized the role of word-of-mouth communication in encouraging screening participation. When a group of individuals undergoes screening, their experiences generate a series of informal discussions that foster public engagement and increase awareness about the screening process and its benefits.

*“If a certain group, say 20 or 50 people, undergoes the screening, that’s enough to create word-of-mouth advertising among the public. They’ll talk about it themselves: ‘I did this and got these results.’ Others will ask, ‘Great, where did you do it? How did you get an appointment?’ This way, the information spreads.”* P10

Many women also emphasized the prominent role of community leaders and social influencers in shaping community norms and practices. They believed these influential figures had the power to encourage screening behaviors to become widely accepted and adopted within the community.

*“When it [screening behavior] comes from decision-makers, people whose words are heard, or social influencers, it can make an impact.”*P6

Several participants discussed religious beliefs and teachings as an essential factor influencing their acceptance of the FIT. Within the framework of Islamic beliefs, which emphasize cleanliness and purity in daily life, handling stool samples is viewed as impure and dirty. This perception seemed to make women more reluctant to engage in the screening.

*“In our Islamic faith, the concept of purity is significant. We tend to view such things [handling a stool sample] as impure and dirty.”* P17

### 5. Policy level factors

#### 5.1. Increasing awareness.

All participants emphasized the importance of fostering collective CRC awareness within the community, achieved through well-funded and promoted campaigns. They identified the absence of widespread CRC awareness campaigns as a major barrier to screening.

*“The first thing necessary is knowledge. The more a person understands the importance of the screening and what the results mean, the faster they can decide about undergoing the test.”* P2

Many women pointed to the MOH’s breast cancer and shingles campaigns as successful efforts that effectively reached and influenced the public. They emphasized the need to adopt similar practices, noted for their extensive reach and use of diverse communication channels.

*“You don’t just decide to go all at once; it has to be multifactorial; it has to speak a language everyone understands, just like with the breast cancer campaign. I see breast cancer awareness as a role model in this regard. It made us all live the experience, and even people who might not be at risk or even of the age of screening still feel like they want to be part of it. All communication channels and media devices have been mobilized for this cause.”* P6

Participants deemed education on various aspects of CRC essential. They particularly highlighted the need for information on the test’s availability and safety, the economic benefits of early CRC diagnosis compared to treatment costs, the risk factors for the disease, and its prognosis.

*“There should be an explanation about what the test results will entail. For example, what is the recovery rate? Aside from the recovery and success rates, what might a person face after their results come out, especially if they are positive? What exactly should they do? What are the implications of the result.”* P2

Participants voiced the need for ongoing media campaigns across various communication channels to ensure broad demographic appeal and reach. They suggested leveraging traditional media channels like TV, radio, billboards, and newer platforms such as social media to connect with varied audiences. Videos and direct text messaging were also recommended as an effective way to engage older adults. To increase accessibility and visibility further, participants proposed placing informational booths in highly trafficked public areas, including workplaces, shopping centers, and entertainment venues.

*“Where do we hear about such matters? We hear them in hospitals while sitting in the waiting room, and you might see some educational content on TV. But this doesn’t reach us in our homes; it doesn’t get to our houses. They need to reach people where they are.”* P14

Several women emphasized the importance of disseminating health information through official channels. Government-issued messages, such as those from the MOH, may be perceived as more credible and authoritative due to the Ministry’s recognized role in safeguarding public health.

*“If there were messages from the Ministry of Health, people would accept them more than from other sources.”* P12

Most women expressed a preference for communication that is simple, clear, and reassuring yet rich with factual content. They valued directness and transparency about the realities of CRC, emphasizing the importance of tailoring messages to different age groups and varying levels of comprehension or CRC risk.

*“For me as a person, I’m very data-driven. So, if there were posts that clearly explain how early detection can reduce the incidence and aid in prevention, and if these posts were widespread on social media platforms, that would honestly encourage me a lot.”* P3

Some participants highlighted the strong influence of personal stories in health communication. They believed that narratives from individuals who have experienced CRC are particularly effective, resonating deeply and making the message more relatable than traditional methods.

*“I am drawn to the approach of bringing in people who have gone through or had CRC to talk about their experiences. It may be a bit challenging, but it is indeed impactful. These stories touch people and have a greater impact, resonating more than traditional methods that might not be as engaging.”* P13

#### 5.2. Enhancing healthcare accessibility.

Several women emphasized the need for uniform availability of screening services across all healthcare facilities, not just governmental primary health centers, to increase accessibility and motivate screening participation.

*“When the screening is available in all hospitals that people frequently visit, no one has an excuse not to get screened.”* P2

Some women called for a well-organized health infrastructure that ensures equitable healthcare access for all, regardless of location. They highlighted that navigating healthcare information is challenging in urban areas and even more difficult in remote regions with limited facilities and services.

*“You hear people saying: “Yes, I really want to do an early screening, but where do I go, to whom?” If this is the case in cities, imagine how much worse it might be in remote areas.”* P3

Lastly, women suggested utilizing digital health applications and governmental platforms to enhance CRCS participation by sending automated, personalized reminders to eligible individuals.

*“Here in Saudi Arabia, I think we should utilize platforms like ‘Absher’ and others because they have our personal data, ages, and everything. They could be used to send reminders.”* P3

## Discussion

Our study offers valuable insights into Saudi women’s perspectives on CRCS. We discovered a complex interplay of factors influencing women’s attitudes and willingness to participate in CRCS, highlighting the need for a multifaceted approach to inform effective public health strategies.

We report a general lack of awareness and knowledge about CRC and its screening among Saudi women, consistent with findings from a recent systematic review on CRC awareness in the region [39]. Similar to Middle Eastern and international reports, low awareness of CRC symptoms, risk factors, and screening modalities has been identified as a prominent barrier to screening uptake [31,40,41]. Despite this limited awareness, we noted a generally positive attitude toward screening, driven by strong beliefs in the benefits of early detection and preventive measures. This aligns with results from a national Saudi survey where 73% of 5720 participants expressed willingness to undergo screening [24]. However, competing life demands frequently interfered with prioritizing screening, a barrier also documented in earlier research [23,31,42].

Ambivalence about screening decisions and personal risk perceptions was prevalent among participants. Justifications for this ambivalence included the absence of symptoms, lack of family cancer history, misconceptions about risk, and perceptions of good health. These factors, noted in previous studies [41,42], reflect a crucial misunderstanding: screening is intended to identify at-risk, asymptomatic individuals with no genetic predispositions [40]. Oster et al. explored this phenomenon and its link to procrastination, emphasizing the importance of enhancing screening availability and convenience, particularly for those lacking personal risk perceptions [43].

Negative emotions related to fear of CRC were observed in this study, aligning with findings from diverse international [31,32] and regional [41] contexts where fear of cancer diagnosis and painful screening procedures are commonly cited barriers to screening. However, in our study, fear appeared to play a dual role in women’s decisions regarding CRCS, serving both as a barrier and a motivator. While some individuals avoid screening for fear of bad news, others are driven to take proactive health measures. This complex impact of fear on health behaviors aligns with findings from the UK, where Young et al. observed fear as both an inhibitor and an enabler of screening participation [44]. Healthcare professionals emphasize that effectively managing negative fear is crucial for improving CRCS uptake. They suggest that informative conversations with patients, enhanced program publicity, and community acceptance can alleviate patient anxiety [45].

Participants generally accepted the screening procedure, but feelings of disgust, embarrassment, and discomfort with handling the test sample were prevalent. This finding aligns with previous international findings associating these emotions with CRCS avoidance [31,46]. Dressler et al. recommended providing antibacterial wipes and disposable gloves alongside the FIT kit to alleviate these concerns [42]. Clear instructions and practical demonstrations were also suggested to address concerns about correctly completing the test, which could potentially increase CRCS uptake [47].

Participants expressed skepticism towards the medical profession’s emphasis on preventive screening measures. Previous studies have noted a similar lack of emphasis among Middle Eastern healthcare providers [41]. For instance, a survey in Al-Khobar City found that only 10% of patients aged 40 or older reported receiving CRCS recommendations from their physicians [48]. Conversely, A recent study in Riyadh City found that most primary healthcare physicians were well-informed and actively engaged in recommending CRCS for asymptomatic patients [49]. The variability in physician engagement underscores the need for further investigation into the consistency of CRCS recommendations across Saudi Arabia. The influence of healthcare professionals on screening behavior is well-documented [50–52]. We support this association, noting that the extent of influence depends on patients’ trust in their doctors and medical institutions. Hoeck et al. reinforced this point by highlighting the critical role of a strong patient-doctor relationship in ensuring adherence to screening recommendations [40]. Furthermore, educational programs delivered or endorsed by health professionals have proven effective in increasing screening participation, likely due to patients’ trust in their physicians and their ability to address patient concerns and fears [47].

Our findings emphasize the necessity of increasing awareness and knowledge about CRC and its screening among Saudi women. Participants called for tailored educational interventions that provide balanced, persuasive information through various media channels and community settings, endorsed by trusted entities like the MOH. Educational messages must resonate with the Saudi community, emphasizing the benefits of early detection for asymptomatic individuals, including improved survival rates. Additionally, they should address the specific cultural sensitivities, barriers, and misconceptions identified in this study. Fear appeals in health communication, which highlight the negative outcomes of screening avoidance, are often criticized for provoking defensive behaviors among audiences. Ruiter et al. noted that enhancing perceived screening effectiveness and the individual’s confidence in their ability to participate in screening (self-efficacy) is more important than fear arousal [53].

Participants underscored the impact of social influence on screening decisions. Integrating patient stories in awareness campaigns might reduce aversion or ambivalence toward screening and help normalize discussions about CRCS [52]. Woudstra and Suurmond highlighted that narratives depicting the screening process and peer experiences can enhance self-efficacy and engagement [54]. Similarly, a UK study showed that supplementing standard information with narrative leaflets positively affected screening intentions [55].

Based on the study findings, we recommend implementing family-focused awareness initiatives that emphasize the crucial role of the family in promoting preventive health behaviors. Educational messages should encourage and normalize screening discussions among family members, frame screening as a shared responsibility, and encourage the family’s support in decision-making. Family involvement is particularly vital in raising awareness among older adults and assisting them in navigating and accessing healthcare services. Including CRC survivors, community leaders, and social influencers as advocates for screening can also positively impact the public’s acceptance and uptake of screening. We also recommend involving religious leaders in awareness initiatives to emphasize Islamic principles supporting disease prevention and to address concerns related to purity.

A recent review in Saudi Arabia found that individuals believe overcoming practical barriers to CRCS is achievable with sufficient motivation and awareness [51]. However, Honein-AbouHaidar et al. stress the need for comprehensive strategies addressing logistical challenges, such as scheduling and service delivery issues, and implementing mass media campaigns to raise awareness and enhance participation rates [52]. We support the need for public health efforts addressing both educational and practical barriers to screening. Our findings revealed women’s mistrust in various aspects of the healthcare system, underscoring the need to strengthen public confidence in its quality and accessibility. To address this mistrust, we emphasize the importance of clear and transparent communication, using media platforms to educate and reassure the public about CRCS quality and safety standards. Additionally, regular updates on service accessibility, operational improvements, and appointment scheduling are essential to help individuals understand the healthcare system’s structure and better navigate care.

Given the myriad factors influencing screening decisions identified in this study, a multi-level intervention approach is essential to strengthen the impact of the CRCS program. Implementing a combination of targeted health promotion efforts can expand program reach, improve engagement, and ultimately enhance patient health outcomes.

### Practice implications

The national CRCS program provides free screening to all citizens upon their request. Yet, findings from this study highlight the need for a more structured approach to identify eligible individuals and send invitations and reminders. Women in this study proposed sending reminders through governmental platforms to encourage timely participation in screening programs. Several studies have corroborated the effectiveness of reminder systems in improving patient alertness and increasing screening uptake [47,56,57]. The program should also incorporate a monitoring system to track screening participation and patient outcomes. Public dissemination of program statistics can facilitate transparent communication, build trust, and motivate participation by demonstrating the program’s reach and effectiveness.

To enhance accessibility, the MOH could collaborate with the private healthcare sector to implement the screening program, as women in this study preferred screening in familiar healthcare settings, whether private or public. Additionally, using mobile clinics to reach the public and distribute FIT kits could improve screening access and engender community discussions on CRC screening.

Integrating CRC screening into routine healthcare checkups provided in clinics and home care services can enhance patient awareness and engagement. Furthermore, embedding automated screening prompts in electronic medical records can remind healthcare professionals to discuss and recommend screening. Finally, as healthcare professionals play a crucial role in influencing patient behaviour, established systems should be in place to ensure they receive regular updates on screening guidelines, remain informed about the CRCS program, and proactively recommend screening to eligible patients.

### Strengths and limitations

This study represents the first qualitative exploration of CRCS views among Saudi women of diverse ages and educational backgrounds. A key strength of our study lies in our analytical approach, which deviates from traditional methods that categorize findings as either barriers or facilitators. Using the Social Ecological Model, we have explored how certain factors can hinder and promote CRCS, depending on the context. This approach has deepened our understanding, providing a comprehensive framework to grasp the complexities of CRCS decisions for women.

Several limitations in this study should be highlighted. Social desirability bias is a well-documented limitation in qualitative interviews; therefore, we implemented several measures to minimize its impact. We maintained a neutral stance throughout the interviews, avoiding subjective reactions or expressions of judgment to participants’ answers. Leading questions were avoided, and participants’ confidentiality and anonymity were ensured to encourage honest discussions. Furthermore, there is a potential self-selection bias in the findings, as participants may have had a pre-existing interest in CRC and its screening.

The interviews were exclusively conducted with participants from the Riyadh region, limiting their representativeness across the wider Saudi population. While similar factors influencing screening behaviours have been identified in other regional studies, certain factors may still vary across regions. However, some insights from this study are transferable to women in other Saudi regions, as well as Muslim and Arab women in different countries who share similar cultural values and backgrounds.

Additionally, since most participants had higher education levels, the findings may primarily reflect challenges as perceived by this educational group, potentially leading to a skewed representation with less emphasis on the cultural, practical, and structural barriers encountered by less educated women. Lastly, by focusing exclusively on women, the study did not explore gender differences in screening-related factors, presenting an area for further research.

## Conclusion

Our study highlighted the complexity of screening behavior and underscored the need for a multifaceted approach to promote CRCS effectively. Public awareness is crucial, yet it should be part of a broader strategy that includes tailored individual, organizational, and policy-level interventions to meet community needs. Additionally, it is essential to examine the perspectives and challenges of healthcare providers and policymakers who play pivotal roles in the design and execution of screening programs. Future research should focus on evaluating the implementation of health promotion interventions within the Saudi national screening program and assessing their long-term impact on screening uptake.
